# Supplementary material for: Efficacy of propylthiouracil in the treatment of pregnancy with hyperthyroidism and its effect on pregnancy outcomes: A meta-analysis
Source: PLoS One. 2022 Mar 10;17(3):e0265085. doi: 10.1371/journal.pone.0265085 (PMC8912150; doi:10.1371/journal.pone.0265085)
Supplement: S1 Materials — (DOCX) [file pone.0265085.s001.docx]

Supplementary table1. Search strategy

**Pubmed: 430**

#10 #3 AND #6 AND #9 430

#9 #7 OR #8 51704

#8 (Hyperthyroid[All Fields]) OR (Hyperthyroids[All Fields]) OR (Primary Hyperthyroidism[All Fields]) OR (Hyperthyroidism, Primary[All Fields])

#7 "Hyperthyroidism"[Mesh]

#6 #4 OR #5 5722

#5 (6-Propyl-2-Thiouracil[All Fields]) OR 6 Propyl 2 Thiouracil[All Fields]

#4 propylthiouracil [Mesh]

#3 #1 OR #2 1110387

#2 (Pregnancies[All Fields]) OR Gestation[All Fields]

#1 "Pregnancy"[Mesh]

**Web of science: 145**

#1 AND #2 AND #3 145

#3 ALL=(propylthiouracil) OR (6-Propyl-2-Thiouracil) OR (6 Propyl 2 Thiouracil) 1519

#2 ALL=(hyperthyroidism) OR (Hyperthyroid) OR (Hyperthyroids ) OR (Primary Hyperthyroidism) OR (Hyperthyroidism, Primary) 8200

#1 ALL=(pregnancy) OR (Pregnancies) OR (Gestation) 293337

**EBSCO: 198**

TX ( (propylthiouracil) OR (6-Propyl-2-Thiouracil) OR (6 Propyl 2 Thiouracil) ) AND TX ( (hyperthyroidism) OR (Hyperthyroid) OR (Hyperthyroids) OR (Primary Hyperthyroidism) OR (Hyperthyroidism, Primary) ) AND TX ( (pregnancy) OR (Pregnancies) OR (Gestation) 198

**Cochrance: 6**

#1 AND #2 AND #3

#1 (propylthiouracil) OR (6-Propyl-2-Thiouracil) OR (6 Propyl 2 Thiouracil)

#2 (hyperthyroidism) OR (Hyperthyroid) OR (Hyperthyroids)) OR (Primary Hyperthyroidism) OR (Hyperthyroidism, Primary)

#3 (pregnancy) OR (Pregnancies) OR (Gestation)

**EMBASE: 707**

#1 AND #2 AND #3 707

#1 propylthiouracil OR '6 propyl 2 thiouracil' OR (6 AND propyl AND 2 AND thiouracil) 11094

#2 hyperthyroidism OR hyperthyroid OR hyperthyroids OR (primary AND hyperthyroidism) OR (hyperthyroidism, AND primary) 54973

#3 pregnancy OR pregnancies OR gestation 1101772

**Scopus: 340**

#1 AND #2 AND #3 340

#1 ALL((propylthiouracil) OR (6-Propyl-2-Thiouracil) OR (6 Propyl 2 Thiouracil) ) 21292

#2 ALL((pregnancy) OR (Pregnancies) OR (Gestation) ) 1742763

#3 ALL((hyperthyroidism) OR (Hyperthyroid) OR (Hyperthyroids)) OR (Primary Hyperthyroidism) OR (Hyperthyroidism, Primary)) 90937

**Wanfang: 40**

[All fields: (propylthiouracil) AND All fields: (pregnancy with hyperthyroidism)](http://s.wanfangdata.com.cn/advanced-search/paper?q=%E5%85%A8%E9%83%A8:(%E4%B8%99%E7%A1%AB%E6%B0%A7%E5%98%A7%E5%95%B6)%20and%20%E5%85%A8%E9%83%A8:(%E5%A6%8A%E5%A8%A0%E5%90%88%E5%B9%B6%E7%94%B2%E4%BA%A2)&type=%5b,,%5d)

**CNKI database: 49**

[Theme: (propylthiouracil) AND Theme: (pregnancy with hyperthyroidism)](http://s.wanfangdata.com.cn/advanced-search/paper?q=%E5%85%A8%E9%83%A8:(%E4%B8%99%E7%A1%AB%E6%B0%A7%E5%98%A7%E5%95%B6)%20and%20%E5%85%A8%E9%83%A8:(%E5%A6%8A%E5%A8%A0%E5%90%88%E5%B9%B6%E7%94%B2%E4%BA%A2)&type=%5b,,%5d)

**VIP database: 19**

All fields=propylthiouracil AND All fields=pregnancy with hyperthyroidism
